# Organizational model for a telepathology system

**DOI:** 10.1186/1746-1596-3-S1-S7

**Published:** 2008-07-15

**Authors:** Roberto Mencarelli, Adriano Marcolongo, Alessio Gasparetto

**Affiliations:** 1 Department of Surgical Pathology, Azienda ULSS 18, Rovigo, Italy; 2 General Manager, Azienda ULSS 18, Rovigo, Italy; 3 Department of Information Technology – Azienda ULSS 18, Rovigo, Italy

## Abstract

The technological development of telemedicine has performed important progress, assuming a diagnostic relief role inside of the processes. Among the fields in fast evolution, telepathology is placed among those of greater interest. Up to some years ago, telepathology allowed us to observe at a distance and in real time, histological or cytological slides through the Internet, using a motorized microscope (dynamic telepathology). Currently, telepathology has completed an important step in ahead being possible to digitize completely a slide and to store it. This allows observation of the whole surface of histological or cytological slides remotely with a customary PC, without human intervention (virtual slide). The described systems have exclusive characteristics, so that a "hybrid system" supporting both technologies, turns out to be the best solution applicable in a wide range program. In order to realize the theoretical aspects previously described, we report an organizational model practicable and applicable to a territory in which three hospitals operate. An essential prerequisite in order to arrange an efficient telepathology system turns out to be one structured data transmission network, equipped with elevated guaranteed bandwidth, and one consolidated experience in the registration and management of digital images.

## Introduction

The acquisition of a telepathology system designed for a district territory that comprises three hospitals managed from two different health administrations (Adria and Rovigo) is inserted in a wide plan of local development of telemedicine. The challenge has been planned from the two administrations and fully financed by the Cariparo Foundation.

In order to achieve the implementation of this telepathology system there was a need for hospitals technology structures innovation. In particular: physical and logical structures for data transport, hardware modernization, software development, processes reorganization.

This technological innovation process is essential for developing and offering to citizens high quality and reliable health services. In particular, a telepathology system needs high hardware and network performance for processing, managing and archiving digital slide images.

The main aims, thanks to the introduction of telepathology, can be summarized as follows:

• Increase of service quality offered to patients thanks to qualified second opinions;

• Sharing and discussion of interesting pathological cases for increasing professional ability;

• Creation of a permanent and available scientific repository.

## Methods

We can distinguish three types of telepathology: static telepathology, dynamic telepathology, virtual slide telepathology. In this paper, we analyze the last two for describing how we can implement a "hybrid telepathology system" that takes advantage of the peculiarities of each one. The dynamic telepathology allows exploration, in real time, of the whole slide surface thanks to a robotized remote controlled microscope; this also permits us to change magnification, focus and digitize the interesting portion. The disadvantages regard the necessary presence of a technician for positioning the desired slide within the microscope and the discontinuous digitization in terms of area and magnification. In virtual slide telepathology, the slide is completely digitized and stored in a repository; this permits a single or multiple user consultation, in every time and without human intervention. The virtual slide allows the exploration of the whole slide surface with different magnification. There are many types of devices that can be used for slide digitization and the right option depends on workload and necessary scan rate [1]. Virtual slide telepathology has a a negligible disadvantage [2]: it is not possible to focus the areas that were not correctly acquired. During the scan process, the scanning device takes into account a finite point number for the focusing procedure; each point is characterized by its own focal plane. When the sample surface is irregular, the device uses proprietary algorithms to calculate an "in focus surface" above the slide; this allows an average good focus but locally a less accurate one. During this treatment, it can be noted that this problem is directly correlated with sample analysis type and grows with increasing histological or cytological sample surface irregularity. Another characteristic to take into account is the virtual slide file size; this depends principally on the following parameters: the resolution, the compression ratio, the compression algorithm and the color depth. Considering the resolution used for the histological and cytological slide scan (0,5 μm/pixel – 20×), the scan area maximum dimension (9 cm2), an adequate compression ratio (15:1) and an appropriate compression algorithm (JPEG2000) it can be possible to obtain averagely a 500 MB file even if the non compressed file is approximately 7.5 GB [3,4]. This file size allows, at present, a selected virtual slide storage for scientific archive creation but doesn't permit complete archiving of all slides; in fact, in case of complete archiving the storage device costs would be much too expensive. Certainly, in the future, the cost/byte reduction for storage and an international agreement about the standard for this storage will permit all slides complete archiving, deleting the conservation necessity. This will lead to many advantages regarding clinical data availability and accessibility. In the end, the necessity of an adequate guaranteed data communication bandwidth for sharing virtual slide must be considered. This problem can be solved thanks to the use of an Image Server and the specific image format (image pyramid); this offers the possibility of selective visualization of images in terms of resolution and portion of interest, without the necessity of a complete transfer of virtual slide from Image Server to a local PC. The complete focusing of the whole slide surface is therefore the real problem to solve, while the archiving and the consultation of virtual slides are effectively faced thanks to scalable and high performance storage devices and dedicated Image Server.

There are mainly two types of second opinions: second opinions in real time on frozen section during surgery (intraoperative consultation) and second opinions on histological or cytological slides which require a complex interpretation. Each type of second opinion has specific characteristics that are directly correlated with previous discussion. In the first second opinion type the analyses are conducted on thick sample or with irregular surface (this arises from the methods through which the samples are obtained and the liquid amount contained into the tissue to analyze); for this reason the automatic scanner device (virtual slide telepathology) can't focus correctly on the whole slide surface but with a motorized microscope (dynamic telepathology) it is possible to focalize each sample surface point. Therefore, the dynamic telepathology represents an important tool for second opinion during intraoperative consultation, especially for the presence on the territory of two hospitals with surgery but without a resident pathologist. Regarding histological and cytological slides, the tissue samples are treated for obtaining thin sections; this deletes the trouble regarding focus due to irregular surface, as previously described. In this case the use of virtual slide telepathology is favorable because it allows the visualization of the morphological picture at any time, remotely with a customary PC, without human intervention. This allows one to obtain a second opinion on complex cases from an expert of personal choice and to perform an external quality control.

Based on previous considerations and for obtaining an effective telepathology system, the best choice is a "hybrid system" composed of motorized microscopes, with remote control, and a scanner for slide digitization, in order to achieve the best characteristics from each system without respective disadvantages. This choice has been applied to Rovigo province health structures, in particular Rovigo, Adria and Trecenta hospitals. For storage purposes it has been used a NAS (Network Attached Storage) device (50 TB capacity – 1 Gb/s transfer rate). The previously described system is completely integrated with the CPOE (Computerized Physician Order Entry) based Hospital information System.

Thanks to complete slides digitization and the use of Image Server with high computational performances, it will be possible to apply filters to acquired images or to apply algorithms for calculating interesting quantities (e.g.: the cellular membrane distribution and continuity). These techniques adequately developed, tested and standardized will be the base for Computer Aided Diagnosis (CAD) introduction.

## Conclusion

The implementation of a telepathology "hybrid system" adequately projected and integrated with the Hospital information System, leads to an increase in the service quality offered by the Pathological Anatomy department, principally thanks to: (a) qualified and real time second opinions on frozen section during surgery or histological-cytological consultation on slides which requires a complex interpretation; (b) continuous education process thanks to the sharing of interesting virtual slides and creation of a permanent scientific archive.

The design and implementation of a telepathology system must be based on real operative demands, supported by a technological structure that can guarantee a reliable and efficient service and projected using hospital experience on digital images acquisition and storage.
